# Differences in overall survival for invasive epithelial ovarian cancer by race and ethnicity: results from the Ovarian Cancer Association Consortium

**DOI:** 10.1038/s41416-026-03509-8

**Published:** 2026-06-30

**Authors:** Nicola S. Meagher, Kami K. White, Lynne R. Wilkens, Hoda Anton-Culver, Elisa V. Bandera, Michael E. Carney, Daniel W. Cramer, Kara L. Cushing-Haugen, Anna DeFazio, Jennifer A. Doherty, Aleksandra Gentry-Maharaj, Ellen L. Goode, Marc T. Goodman, Holly R. Harris, Beth Y. Karlan, Scott H. Kaufmann, Linda E. Kelemen, Martin Köbel, Jennifer M. Koziak, Loic Le Marchand, Jenny Lester, Valerie McGuire, Usha Menon, Francesmary Modugno, Kirsten B. Moysich, Celeste Leigh Pearce, Paul D. P. Pharoah, Malcolm C. Pike, Bo Qin, Harvey A. Risch, Joseph H. Rothstein, Danja Sarink, Weiva Sieh, Helen Steed, Kathryn L. Terry, Pamela J. Thompson, Linda J. Titus, Toon Van Gorp, Alice S. Whittemore, Stacey J. Winham, Susan J. Jordan, Anna H. Wu, Penelope M. Webb, Lauren C. Peres, Melissa A. Merritt

**Affiliations:** 1 The Daffodil Centre, The University of Sydney and Cancer Council NSW, Sydney, NSW Australia; 2 Medicines Intelligence Program, School of Population Health, UNSW Medicine and Health, University of NSW, Sydney, NSW Australia; 3 Population Sciences in the Pacifi c Program (Cancer Epidemiology), University of Hawaii Cancer Center, Honolulu, HI USA; 4 Department of Medicine, Genetic Epidemiology Research Institute, University of California Irvine, Irvine, CA USA; 5 Cancer Prevention and Control Program, Rutgers Cancer Institute, New Brunswick, NJ USA; 6 Department of Obstetrics and Gynecology, John A. Burns School of Medicine, University of Hawaii, Honolulu, HI USA; 7 Obstetrics and Gynecology Epidemiology Center, Department of Obstetrics and, Brigham and Women’s Hospital and Harvard Medical School, Boston, MA USA; 8 Department of Epidemiology, Harvard TH Chan School of Public Health, Boston, MA USA; 9 Program in Epidemiology, Division of Public Health Sciences, Fred Hutchinson Cancer Center, Seattle, WA USA; 10 Centre for Cancer Research, The Westmead Institute for Medical Research, University of Sydney, Sydney, NSW Australia; 11 Faculty of Medicine and Health, The University of Sydney, Sydney, NSW Australia; 12 Department of Gynaecological Oncology, Westmead Hospital, Sydney, NSW Australia; 13 Department of Population Health Sciences, Huntsman Cancer Institute, University of Utah, Salt Lake City, UT USA; 14 Department of Women’s Cancer, Elizabeth Garrett Anderson Institute for Women’s Health, University College London, London, UK; 15 Department of Quantitative Health Sciences, Division of Epidemiology, Mayo Clinic, Rochester, MN USA; 16 Cancer Prevention and Control Program, Cedars-Sinai Cancer, Cedars-Sinai Medical Center, Los Angeles, CA USA; 17 Department of Epidemiology, University of Washington, Seattle, WA USA; 18 David Geffen School of Medicine, Department of Obstetrics and Gynecology, University of California at Los Angeles, Los Angeles, CA USA; 19 Departments of Medicine and Molecular Pharmacology, Mayo Clinic, Rochester, MN USA; 20 Communicable Disease Epidemiology Section, South Carolina Department of Public Health, Columbia, SC USA; 21 Department of Pathology and Laboratory Medicine, University of Calgary, Calgary, AB Canada; 22 Alberta Health Services-Cancer Care, Calgary, AB Canada; 23 Department of Epidemiology and Population Health, Stanford University School of Medicine, Stanford, CA USA; 24 UCL Innovative Clinical Trials Unit, Institute of Clinical Trials and Methodology, University College London, London, UK; 25 Department of Obstetrics, Gynecology and Reproductive Sciences, Division of Gynecologic Oncology, University of Pittsburgh School of Medicine, Pittsburgh, PA USA; 26 Department of Epidemiology, University of Pittsburgh School of Public Health, Pittsburgh, PA USA; 27 Women’s Cancer Research Center, Magee-Womens Research Institute and Hillman Cancer Center, Pittsburgh, PA USA; 28 Department of Cancer Prevention and Control, Roswell Park Cancer Institute, Buffalo, NY USA; 29 Department of Epidemiology, University of Michigan School of Public Health, Ann Arbor, MI USA; 30 Department of Computational Biomedicine, Cedars-Sinai Medical Center, West Hollywood, CA USA; 31 Department of Epidemiology and Biostatistics, Memorial Sloan-Kettering Cancer Center, New York, NY USA; 32 Department of Population Health and Public Health Sciences, Keck School of Medicine, University of Southern California Norris Comprehensive Cancer Center, Los Angeles, CA USA; 33 Chronic Disease Epidemiology, Yale School of Public Health, New Haven, CT USA; 34 Department of Epidemiology, University of Texas MD Anderson Cancer Center, Houston, TX USA; 35 Department of Population Health Science and Policy, Icahn School of Medicine at Mount Sinai, New York, NY USA; 36 Department of Obstetrics and Gynecology, University of Alberta, Edmonton, AB Canada; 37 Samuel Oschin Comprehensive Cancer Institute, Cancer Prevention and Genetics Program, Cedars-Sinai Medical Center, Los Angeles, CA USA; 38 Dartmouth Cancer Center, Lebanon, NH USA; 39 Division of Gynaecologic Oncology, Leuven Cancer Institute, University Hospital Leuven and KU Leuven, Leuven, Belgium; 40 Department of Biomedical Data Science, Stanford University School of Medicine, Stanford, CA USA; 41 Department of Quantitative Health Sciences, Division of Computational Biology, Mayo Clinic, Rochester, MN USA; 42 School of Public Health, The University of Queensland, Brisbane, QLD Australia; 43 Population Health Program, QIMR Berghofer Medical Research Institute, Brisbane, QLD Australia; 44 Peter MacCallum Cancer Centre, Melbourne, VIC Australia; 45 Department of Cancer Epidemiology, Moffitt Cancer Center, Tampa, FL USA

**Keywords:** Cancer epidemiology, Ovarian cancer

## Abstract

**Background:**

Prior studies have examined survival in patients with epithelial ovarian cancer (EOC); however, few consider race and ethnicity, particularly disaggregating Asian and Native Hawaiian/Pacific Islander women.

**Methods:**

We analysed data from 18 Ovarian Cancer Association Consortium studies, including women with EOC from Asian (*n* = 697), non-Hispanic Black (*n* = 267), Hispanic (*n* = 492), Native Hawaiian/Pacific Islander (*n* = 98) and non-Hispanic White (*n* = 12,998) racial and ethnic groups. We ran Cox proportional hazards models estimating overall survival by race and ethnicity, adjusting for age, stage, year of diagnosis, and histotype, with fully adjusted models accounting for body mass index, smoking, and postmenopausal hormone use. We also examined associations between hormone-related factors and family history and overall survival by race and ethnicity, testing for heterogeneity.

**Results:**

Compared to non-Hispanic White women with EOC, Native Hawaiian/Pacific Islander and non-Hispanic Black women had poorer overall survival (Hazard Ratios, HR = 1.58, 95% CI = 1.16–2.16, and HR = 1.31, 95% CI = 1.12–1.54, respectively). The association was more pronounced for Native Hawaiian/Pacific Islander women with high-grade serous carcinoma (HR = 2.00, 95% CI = 1.37–2.92). There was no significant heterogeneity in the associations between epidemiological factors and survival by racial and ethnic groups (*p* ≥ 0.31).

**Discussion:**

Native Hawaiian/Pacific Islander and non-Hispanic Black women with EOC had poorer survival, highlighting the need to address disparities in outcome.

## Background

Ovarian cancer is the 8th most common cancer diagnosed in women globally, with variable age-adjusted incidence rates across different countries [1]. Survival varies by race and ethnicity, and is notably worse for Black women with epithelial ovarian cancer (EOC) compared with White women, as shown by the Ovarian Cancer in Women of African Ancestry (OCWAA) consortium [2]. Other studies have shown that, on average, Asian [3, 4] and Hispanic [5] women with EOC have better or equivalent survival, respectively, compared to non-Hispanic White women. A recent analysis using 2006–2020 Surveillance, Epidemiology and End Results (SEER) registry data found that compared to non-Hispanic White women, Native Hawaiian/Pacific Islander women with EOC had significantly poorer survival [6]. Asian and Native Hawaiian/Pacific Islander individuals have historically been grouped in mortality data and health statistics in the US, which may have masked disparities between these groups [7].

Established clinical and pathological factors associated with EOC survival include histotype, stage, and residual disease following primary surgery [8]. Prior epidemiological studies have shown associations between pre-diagnosis lifestyle factors with survival in EOC, such as poorer survival for current and former smokers compared with non-smokers [9, 10] and worse survival for EOC cases with obesity compared with cases with a lean body mass index (BMI) [11, 12]. Ever use of postmenopausal hormones prior to an EOC diagnosis has been associated with improved ovarian cancer-specific survival [13–15], but no association with survival has been observed for other reproductive factors such as oral contraceptive (OC) use, age at menarche, parity or breastfeeding [15, 16]. The current study examined differences in overall survival across Asian, non-Hispanic Black, Hispanic, Native Hawaiian/Pacific Islander, and non-Hispanic White women with EOC. Additionally, given some of the established differences in survival across racial and ethnic groups, we evaluated associations between hormone-related factors and family history with survival by racial and ethnic group.

## Methods

### Study population

The study population (*n* = 14,679 cases with invasive EOC) was drawn from 18 Ovarian Cancer Association Consortium (OCAC) studies based in Australia, Belgium, Canada, England, and the USA. Studies were eligible for this analysis if they collected extensive epidemiological risk factor data, had data on overall survival (death from any cause) and had 10 or more ovarian cancer cases with a self-reported Asian, Native Hawaiian/Pacific Islander, non-Hispanic Black or Hispanic race or ethnicity.

Racial and ethnic groups in the original OCAC database were based on self-reports from questionnaires for all study participants. Since Asian and Native Hawaiian/Pacific Islander participants (*n* = 922) were combined into one group in the original database, detailed self-reported data on Asian and Native Hawaiian/Pacific Islander race from questionnaires were requested from selected studies with the largest representation of these subjects: Diseases of the Ovary and their Evaluation Study, DOV; Hawaii Ovarian Cancer Case-Control Study, HAW; Genetic Epidemiology of Ovarian Cancer, STA; and Los Angeles County Case-Control Studies of Ovarian Cancer, USC. Thus, classification of Asian and Native Hawaiian/Pacific Islander participants used these self-reported data (for *n* = 521 and 93 subjects, respectively) and, when not available, used genetic ancestry data (*n* = 119 and five subjects, respectively). Genetic ancestry was determined based on clusters created from the Oncoarray principal component analysis (PCAs) and/or the Collaborative Oncological Gene-Environment Study (COGS) PCAs; 67 participants had Oncoarray and COGS values (all concordant), and 57 participants had either Oncoarray or COGS data [17, 18]. Using these data, not all participants in the combined Asian and Native Hawaiian/Pacific Islander participants could be disaggregated further, therefore these individuals (*n* = 127) were reported as “not specified”.

### Ethics approval and consent to participate

Approval for this study was obtained from the University of Hawaii (Protocol # 2019-00087). All participants provided either written informed consent or implicit consent through return of the study questionnaire. Participating studies obtained institutional review board (IRB) approval at their respective institutions, and the OCAC Coordinating Center (Duke University) received IRB approval from its institution and participating registries as required for data acquisition, pooling, and harmonisation. All methods were performed in accordance with the relevant guidelines and regulations.

### Epidemiological variables

Covariates that were available from the questionnaires included: age at EOC diagnosis, age at menarche, duration of OC use, parity, breastfeeding among parous women, menopausal status, and postmenopausal hormone use (referring to ever use of estrogen only, estrogen plus progesterone and unknown formulation types), endometriosis, tubal ligation or hysterectomy (at least one year prior to EOC diagnosis date), recent BMI (defined as one year prior to the diagnosis date for all study sites except for the CON, DOV, HAW and OPL, where BMI referred to five years prior to the diagnosis date), smoking and first-degree family history of breast cancer or ovarian cancer.

Clinical and pathological variables included: stage (localised, regional, distant, unknown); and histotype (high-grade serous, low-grade serous, endometrioid, mucinous, clear cell or other epithelial). High-grade serous tubo-ovarian carcinoma (HGSC) was defined as serous histology grades 2–4; serous histology with missing/unknown grade; endometrioid histology grades 3–4 [to avoid misclassification of HGSC as endometrioid [19, 20]]; or poorly differentiated epithelial histology grades 2–4. Information on treatment received or residual disease after surgery was not available in the analytic dataset. Supplementary Table S1 shows the information collected by some consortium studies and the high level of missingness in this dataset, which meant that these data were not used in the current analysis.

### Statistical analysis

Cox proportional hazards regression models were used to estimate hazard ratios (HR) and 95% confidence intervals (95% CI) for overall survival. Overall survival time was calculated as the number of days from diagnosis to last follow-up or death from any cause, whichever occurred first. Left truncation was used to account for days from diagnosis to study enrolment to reduce the chance of survivorship bias arising from the exclusion of eligible women who died before they had an opportunity to be enrolled. Stage and histotype did not meet the proportional hazards assumption. To address this, stage was modelled as a strata term. We included an interaction term for histotype and time, however this did not appreciably change the associations, therefore this interaction term was not included in the final models. We also assessed additional adjustment for education level; however, this did not change the results (<10% HR change), and therefore education was not included in the final models.

A multivariable model was adjusted for age and year at diagnosis (continuous), histotype [HGSC (reference), low-grade serous, endometrioid, mucinous, clear cell and other epithelial] and included strata variables for study site, age at diagnosis (10-year groups) and stage (localised, regional, distant, unstaged/missing). The addition of age as a continuous variable was included in the model to account for any imbalance in the 10-year age categories across racial and ethnic groups. A fully adjusted model included the same factors listed above plus adjustment for additional factors associated with EOC survival in the literature and which could plausibly vary across racial and ethnic groups: recent BMI [<25 kg/m^2^ (reference), 25.0-29.9, ≥30.0], smoking [never (reference), former, current] and any postmenopausal hormone use [never (reference), ever, pre/peri-menopausal]. The models examined overall survival with race and ethnicity as the exposure [six groups; non-Hispanic White (reference), Hispanic, non-Hispanic Black, Asian, Native Hawaiian/Pacific Islander, and Asian or Native Hawaiian/Pacific Islander not specified]. Sensitivity analyses were conducted on more focused disease groups, specifically HGSC, the most common histological subtype of EOC, and advanced stage (regional and distant) disease to reduce heterogeneity.

We next evaluated associations for hormone-related factors and family history with overall survival across six racial and ethnic groups. Interactions between race and ethnicity (six groups) with each exposure of interest were evaluated by comparing multivariable models with and without interaction terms to calculate p-values for heterogeneity using the global Wald test. Tests for linear trend (p for trend) were performed using variables based on the median values for each category when applicable. All analyses were performed using SAS version 9.4.

## Results

**Fig. 1:**
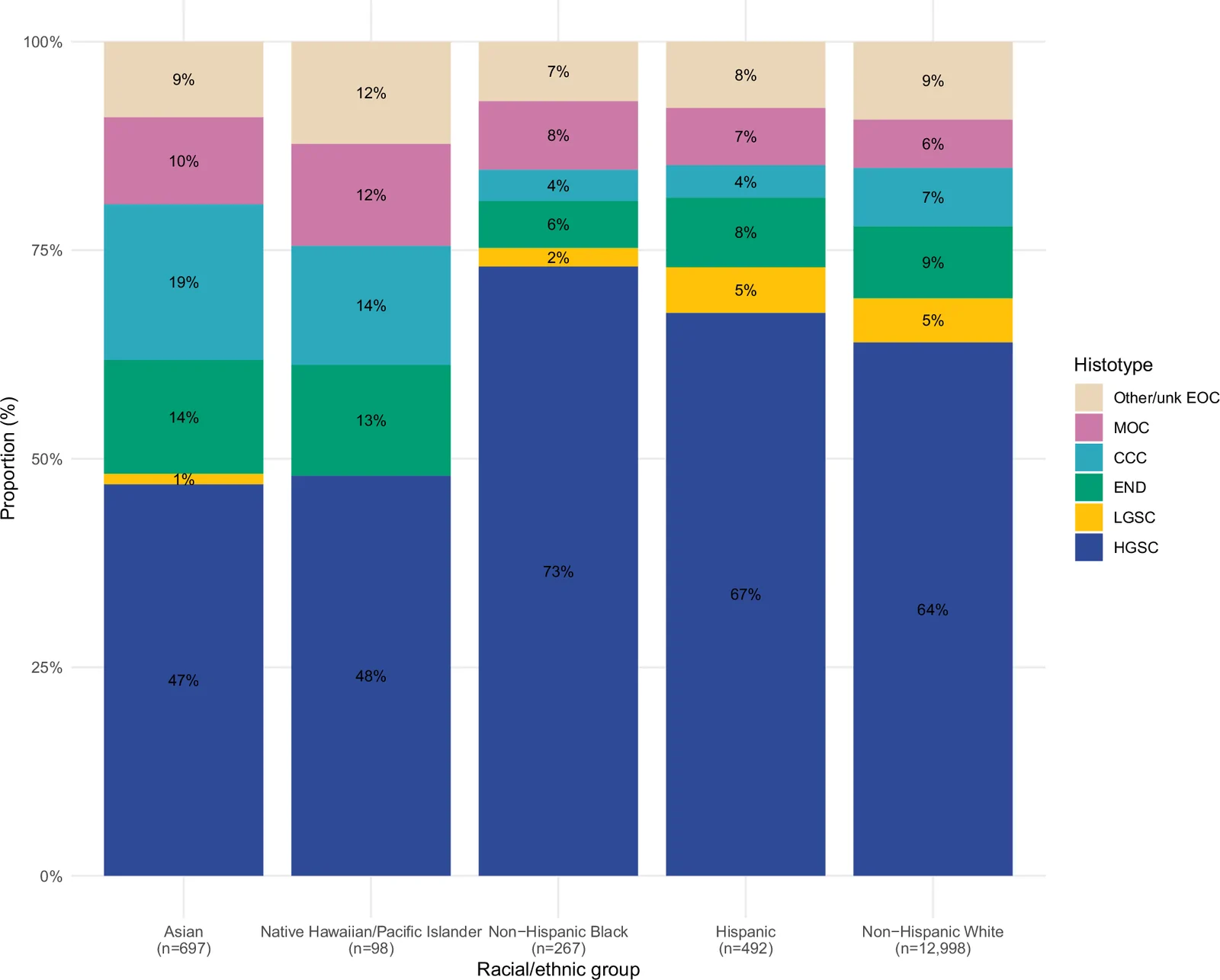
Frequency of epithelial ovarian cancer histotypes by racial and ethnic group. There were no cases of LGSC in the Native Hawaiian/Pacific Islander group. Abbreviations: CCC (clear cell carcinoma); END (endometrioid carcinoma); HGSC (high-grade serous tubo-ovarian carcinoma); LGSC (low-grade serous carcinoma); MOC (mucinous ovarian carcinoma). HGSC includes serous histology grades 2–4; serous histology with missing/unknown grade; endometrioid histology grade 3–4 and poorly differentiated epithelial histology grades 2–4. Other epithelial includes mixed cell, unknown or other epithelial.

In total, 58% of the 14,679 patients with invasive EOC died (any cause death) during the study period (Table 1), with a median follow-up time for patients who were alive during the study period from 2 to 15 years after diagnosis. Patients were diagnosed between 1968 and 2018, and the overall median (interquartile range) year of diagnosis across studies was 2004 (2001–2007), Supplementary Table S2. There were some studies representing the majority of participants from selected racial and ethnic groups, for example, 87% of Native Hawaiian/Pacific Islander participants were from the Hawaii Ovarian Cancer Case Control Study (HAW study), and 59% of Hispanic participants and 46% of non-Hispanic Black participants came from the Los Angeles County Case-Control Studies of Ovarian Cancer, USC. There were notable differences in the age at diagnosis, stage and histotype distributions across racial and ethnic groups (Table 2). Compared with non-Hispanic White participants, all other racial and ethnic groups were younger at diagnosis. Non-Hispanic Black, Hispanic and non-Hispanic White women had the highest proportion of HGSC tumours (≥64%) while HGSC comprised ~48% for Asian and Native Hawaiian/Pacific Islander women (Fig. 1). Notably, clear cell carcinoma was most frequent in Asian and Native Hawaiian/Pacific Islander women (19 and 14%, respectively). Within Native Hawaiian/Pacific Islander women from the HAW study, the frequency of clear cell carcinoma was similar at 12% (data not shown). Approximately two-thirds of non-Hispanic Black, non-Hispanic White women and Hispanic women were diagnosed with distant stage disease, compared with less than half of Asian and Native Hawaiian/Pacific Islander women (Table 2), in line with the histotype distribution of less HGSC cases.

**Table 1:** Description of the 18 OCAC studies included in the analysis of racial and ethnic differences in invasive epithelial ovarian cancer survival.

Study^a^	Cases *N*	Deaths *N*	Overall survival for alive, years^b^	Overall survival for deceased years^b^	Age at diagnosis, years	Asian	Native Hawaiian/ Pacific Islander	Asian or Native Hawaiian/ Pacific Islander, not specified^c^	Non-Hispanic Black	Hispanic	Non-Hispanic White
Total	14,679	8571				697	98	127	267	492	12,998
AOV	277	144	7.4 (5.5, 12.7)	2.8 (1.3, 5.3)	55 [48–66]	57 (8.2)	0	0	1 (0.4)	0	219 (1.7)
AUS	1540	1107	8.6 (5.5, 10.5)	2.9 (1.6, 5.1)	60 [52–68]	36 (5.2)	3 (3.1)	16 (8.7)	0	0	1485 (11.4)
BEL	621	400	4.8 (2.8, 8.8)	3.5 (2.2, 5.4)	59 [50–67]	1 (0.8)	1 (1.0)	6 (3.3)	2 (0.7)	1 (0.2)	610 (4.7)
CON	385	222	8.3 (7.5, 9.5)	3.2 (2.1, 5.1)	59 [52–68]	0	0	3 (1.6)	8 (3.0)	6 (1.2)	368 (2.8)
DOV	1125	683	11.6 (9.6, 13.7)	3.6 (2.1, 5.5)	57 [49–63]	57 (8.2)	6 (6.1)	0	12 (4.5)	35 (7.1)	1015 (7.8)
HAW	424	239	11.0 (8.5, 15.6)	3.6 (2.1, 6.5)	56 [48–66]	228 (32.7)	85 (86.7)	0	0	7 (1.4)	104 (0.8)
HOP	709	428	8.3 (6.4, 9.4)	3.0 (1.8, 4.7)	59 [51–69]	1 (0.1)	0	2 (1.1)	23 (8.6)	2 (0.4)	681 (5.2)
LAX	352	225	5.7 (4.2, 9.3)	3.0 (1.8, 4.8)	58 [49–67]	12 (1.7)	0	4 (2.2)	18 (6.7)	7 (1.4)	311 (2.4)
MAY	1463	804	4.3 (1.8, .8.2)	2.7 (1.5, 4.6)	62 [54–71]	4 (0.6)	0	7 (3.8)	7 (2.6)	9 (1.8)	1436 (11.0)
MSK	505	151	1.9 (0.7, 4.1)	2.3 (1.2, 3.6)	60 [53–69]	24 (3.4)	0	9 (4.9)	22 (8.2)	23 (4.7)	427 (3.3)
NEC	1255	844	8.8 (3.9, 12.4)	3.6 (2.2, 5.8)	56 [49–65]	10 (1.4)	0	10 (5.4)	19 (7.1)	17 (3.5)	1199 (9.2)
NJO	235	141	9.0 (7.7, 9.7)	3.8 (2.5, 6.0)	56 [50–62]	2 (0.3)	0	7 (3.8)	9 (3.4)	11 (2.2)	206 (1.6)
OPL	798	459	6.8 (5.9, 7.5)	3.2 (1.9, 4.6)	61 [52–68]	8 (1.2)	1 (1.0)	41 (22.3)	0	0	748 (5.8)
SEA	1469	648	6.6 (3.2, 12.5)	4.5 (2.9, 6.9)	58 [50–64]	3 (0.4)	0	8 (4.3)	2 (0.8)	0	1456 (11.2)
STA	448	242	11.0 (9.9, 12.2)	3.4 (2.1, 4.8)	51 [44–58]	79 (11.3)	2 (2.0)	0	17 (6.4)	47 (9.6)	303 (2.3)
UCI	429	205	8.8 (7.7, 10.3)	4.4 (2.9, 6.8)	58 [50–68]	16 (2.3)	0	8 (4.3)	0	36 (7.3)	369 (2.8)
UKO	743	393	8.7 (7.4, 11.3)	3.4 (1.8, 5.4)	60 [53–68]	1 (0.1)	0	6 (3.3)	4 (1.5)	0	732 (5.6)
USC	1901	1233	15.0 (9.7, 19.5)	3.6 (2.0, 7.0)	58 [49–66]	158 (22.7)	0	0	123 (46.1)	291 (59.2)	1329 (10.2)

Values are medians (IQR) for continuous variables or *N* (%) for categorical variables.

IQR (interquartile range), OCAC (Ovarian Cancer Association Consortium).

^a^Study acronyms and years of interview: AOV (Alberta Ovarian Tumor Types study, Canada, 1978–2010, PubMed ID: 25349970); AUS (Australian Ovarian Cancer and Australian Cancer Study, 2002–2005, PubMed ID:17721999); BEL (University Hospitals Leuven, Department of Gynaecological Oncology, Tumor biobank, Belgium, 2007–2017); CON (Connecticut Ovary Study, 1999–2003 PubMed ID:16985038); DOV (Diseases of the Ovary and their Evaluation Study, 2002–2009, PubMed ID: 23065074); HAW (Hawaii Ovarian Cancer Case-Control Study, 1993–2008, PubMed ID:18667686); HOP (Hormones and Ovarian Cancer PrEdiction, 2003–2009, PubMed ID: 22252409); LAX (Women’s Cancer Program at the Samuel Oschin Comprehensive Cancer Institute, 1989–2009, PubMed ID: 22253144); MAY (Mayo Clinic Ovarian Cancer Case Control Study, 2000–2008, PubMed ID:18381459); MSK (Memorial Sloan Kettering Cancer Center Gynecologic Tissue Bank, 1997–2010); NEC (New England Case-Control Study of Ovarian Cancer, 1992–2008, PubMed ID:15994977); NJO (New Jersey Ovarian Cancer Study, 2002–2008, PubMed ID:21943063); OPL (Ovarian Cancer Prognosis and Lifestyle study, Australia, 2012–2015, PubMed ID: 33749900); SEA (UK Studies of Epidemiology and Risk Factors in Cancer Heredity (SEARCH) Ovarian Cancer Study, 1998–2016, PubMed ID: 16774946); STA (Genetic Epidemiology of Ovarian Cancer, 1998–2016, PubMed ID:15383404); UCI (University of California, Irvine Ovarian Cancer Study, 1995–2005, PubMed ID:10667470); UKO (United Kingdom Ovarian Cancer Population Study, 2006–2010, PubMed ID: 21074968); USC (Los Angeles County Case-Control Studies of Ovarian Cancer, 1993–2010, PubMed ID:11821246).

^b^Overall survival time was calculated from the date of diagnosis to the date of last follow-up or death.

^c^Asian or Native Hawaiian/Pacific Islander, not specified, includes individuals who were unable to be classified into more specific groups.

**Table 2:** Characteristics of invasive epithelial ovarian cancer cases by racial and ethnic group^a^.

	Asian (*n* = 697)	Native Hawaiian/Pacific Islander (*n* = 98)	Asian or Native Hawaiian/Pacific Islander, not specified (*n* = 127)^f^	Non-Hispanic Black (*n* = 267)	Hispanic (*n* = 492)	Non-Hispanic White (*n* = 12,998)
Vital status						
Alive	369 (52.9)	39 (39.8)	63 (49.6)	95 (35.6)	207 (42.1)	5335 (41.0)
Deceased	328 (47.1)	59 (60.2)	64 (50.4)	172 (64.4)	285 (57.9)	7663 (59.0)
Age at diagnosis (years)						
<40	82 (12.2)	15 (15.3)	27 (21.3)	17 (6.4)	47 (9.6)	612 (4.7)
40–49	201 (28.8)	18 (18.4)	35 (27.6)	67 (25.1)	116 (23.6)	2064 (15.9)
50–59	195 (28.0)	37 (37.8)	34 (26.8)	85 (31.8)	163 (33.1)	4056 (31.2)
60–69	131 (18.8)	20 (20.4)	24 (18.9)	68 (25.5)	118 (24.0)	4008 (30.8)
≥70	88 (12.6)	8 (8.2)	7 (5.5)	30 (11.2)	48 (9.8)	2258 (17.4)
Age at menarche (years)						
<12	120 (20.0)	28 (28.9)	20 (18.2)	66 (28.7)	114 (24.8)	2260 (19.5)
12–13	286 (47.6)	47 (48.5)	58 (52.7)	109 (47.4)	234 (50.9)	6145 (52.9)
≥14	195 (32.5)	22 (22.7)	32 (29.1)	55 (23.9)	112 (24.4)	3203 (27.6)
Missing	96	1	74	37	32	1390
Education						
<High school	81 (13.9)	16 (16.5)	8 (7.8)	22 (12.0)	123 (34.8)	1323 (12.7)
≥High school	500 (86.1)	81 (83.5)	94 (92.2)	162 (88.0)	230 (65.2)	9099 (87.3)
Missing	116	1	25	83	139	2576
OC use						
Never	384 (64.2)	58 (59.2)	59 (55.1)	86 (38.9)	231 (50.3)	4617 (39.9)
<5 years	148 (24.8)	29 (29.6)	33 (30.8)	92 (41.6)	155 (33.8)	3674 (31.8)
≥5 years	66 (11.0)	11 (11.2)	15 (14.0)	43 (19.5)	73 (15.9)	3273 (28.3)
Missing	99	0	20	46	33	1434
Parity						
0 live births	227 (34.2)	24 (24.7)	33 (29.0)	43 (18.1)	89 (19.2)	2737 (22.6)
1	108 (16.3)	13 (13.4)	21 (8.4)	42 (17.7)	71 (15.3)	1652 (13.6)
2	162 (24.4)	27 (27.8)	38 (33.3)	46 (19.4)	106 (22.9)	3901 (32.1)
≥3	166 (25.0)	33 (34.0)	22 (19.3)	106 (44.7)	197 (42.6)	3846 (31.7)
Missing	34	1	13	30	29	862
Tubal ligation						
No	530 (87.5)	84 (85.7)	93 (88.6)	186 (79.2)	374 (81.8)	9042 (83.3)
Yes	76 (12.5)	14 (14.3)	12 (11.4)	49 (20.9)	83 (18.2)	1812 (16.7)
Missing	91	0	22	32	35	2144
Breastfeeding^b^						
No	105 (27.7)	23 (31.9)	9 (13.4)	74 (48.7)	103 (36.3)	2726 (37.1)
Yes	274 (72.3)	49 (68.1)	58 (86.6)	78 (51.3)	181 (63.7)	4620 (62.9)
Missing	66	2	16	50	93	2462
Menopausal status						
Pre/peri	289 (43.8)	32 (33.0)	56 (49.2)	79 (33.3)	161 (35.0)	3074 (25.4)
Post	371 (56.2)	65 (67.0)	58 (50.8)	158 (66.7)	299 (65.0)	9048 (74.6)
Missing	37	1	13	30	32	876
Postmenopausal hormone use^c^						
Never	192 (57.3)	52 (80.0)	32 (61.5)	92 (65.7)	186 (63.3)	4476 (54.9)
Ever	143 (42.7)	13 (20.0)	20 (38.5)	48 (34.3)	108 (36.7)	3678 (45.1)
Missing	36	0	64	18	5	894
Endometriosis						
No	465 (85.8)	90 (93.8)	79 (79.8)	180 (89.1)	378 (92.7)	8620 (89.1)
Yes	77 (14.2)	6 (6.3)	20 (20.2)	22 (10.9)	30 (7.4)	1057 (10.9)
Missing	155	2	28	65	84	3321
Hysterectomy^d^						
No	547 (90.3)	89 (90.8)	92 (92.0)	166 (74.4)	367 (82.5)	8275 (79.5)
Yes	59 (9.7)	9 (9.2)	8 (8.0)	57 (25.6)	78 (17.5)	2129 (20.5)
Missing	91	0	27	44	47	2594
BMI^e^						
<25.0 kg/m^2^	335 (65.3)	25 (26.6)	68 (26.6)	61 (29.1)	153 (37.3)	4302 (46.2)
25.0–29.9	126 (24.6)	32 (34.0)	21 (21.4)	76 (36.2)	132 (32.2)	2749 (29.6)
≥30.0	52 (10.1)	37 (39.4)	9 (9.2)	73 (34.8)	125 (30.5)	2252 (24.2)
Missing	184	4	29	57	82	3695
Smoking						
Never	533 (43.2)	59 (60.8)	87 (82.9)	83 (44.6)	218 (61.2)	5876 (54.4)
Former	27 (4.2)	14 (14.4)	4 (3.8)	59 (31.7)	97 (27.3)	3606 (33.4)
Current	81 (12.6)	24 (24.7)	14 (13.3)	44 (23.7)	41 (11.5)	1329 (12.3)
Missing	56	1	22	81	136	2187
Family history of breast cancer						
No	348 (86.1)	39 (69.6)	92 (87.6)	153 (84.8)	309 (84.2)	8387 (80.6)
Yes	56 (13.9)	17 (30.4)	13 (12.4)	37 (19.5)	58 (15.8)	2013 (19.4)
Missing	293	42	22	77	125	2598
Family history of ovarian cancer						
No	364 (95.0)	47 (94.0)	102 (98.1)	168 (92.3)	332 (92.2)	9580 (93.8)
Yes	19 (5.0)	3 (6.0)	2 (1.9)	14 (7.7)	28 (7.8)	631 (6.2)
Missing	314	48	23	85	132	2787
Stage						
Localized	209 (30.3)	33 (33.7)	16 (13.3)	44 (17.1)	89 (18.4)	1971 (16.0)
Regional	156 (22.6)	20 (20.4)	30 (35.0)	29 (11.2)	78 (16.2)	2102 (17.1)
Distant	324 (47.0)	45 (45.9)	74 (61.7)	185 (71.7)	316 (65.4)	8233 (66.9)
Unknown stage	84	0	7	9	9	692

Values are *N* (%), percentages are for non-missing data.

BMI (Body Mass Index), OC (Oral Contraceptive).

^a^The following variables were missing for certain study sites: breastfeeding (AOV, MAY); endometriosis (STA); BMI recent (AOV, STA); BMI at age 18 (AOV, MAY, STA); postmenopausal hormone use (AOV, STA); all of the following variables were missing: education, OC use, family history of breast or ovarian cancer, hysterectomy and tubal ligation (AOV).

^b^Breastfeeding refers to parous women only.

^c^Postmenopausal hormone use refers to postmenopausal women only.

^d^Hysterectomy analysis excludes one study outlier (MAY, all values set to missing).

^e^BMI refers to one year before the diagnosis date for all sites, except for CON, DOV, HAW and OPL (five years before the diagnosis date).

^f^Asian or Native Hawaiian/Pacific Islander not specified refers to individuals who were unable to be classified into more specific groups.

When comparing the distribution of epidemiological risk factors across racial and ethnic groups, ever use of postmenopausal hormones was least frequent in Native Hawaiian/Pacific Islander women (20% compared with 34–45% use in other groups) (Table 2). High BMI was most prevalent in the Native Hawaiian/Pacific Islander group, 39% had a BMI ≥ 30 kg/m^2^, compared with 10% of Asian women in this BMI category. More than half of non-Hispanic Black women (55%) reported former or current smoking, and this frequency was lowest in Asian women (17%). A family history of breast cancer was reported in a third of Native Hawaiian/Pacific Islander women, slightly less in the other groups (≤20%), and a family history of ovarian cancer was similar across all racial and ethnic groups (<8%).

Compared with non-Hispanic White women with EOC, Native Hawaiian/Pacific Islander, and non-Hispanic Black women had poorer overall survival (HR = 1.58, 95% CI = 1.16–2.16 and HR = 1.31, 95% CI = 1.12–1.54, respectively) in a multivariable model (Model 1) that accounted for age and year of diagnosis, histotype, study and stage (Table 3). There was no statistically significant difference in survival for Hispanic participants (HR = 1.09, 95% CI = 0.96–1.24), however among Asian women compared with non-Hispanic White women, there was a non-significant improvement in survival (HR = 0.89, 95% CI = 0.78, 1.01), which was significant among cases with regional or distant disease (HR = 0.85, 95% CI = 0.74, 0.98). In the fully adjusted model (additional adjustment for BMI, smoking and postmenopausal hormone use, Model 2), HRs for total EOC were slightly attenuated for Native Hawaiian/Pacific Islander (HR = 1.47, 95% CI = 1.06–2.04) and non-Hispanic Black women (HR = 1.23, 95% CI = 0.99–1.51). When we restricted to the subgroup with HGSC and then to those with regional or distant stage, the pattern of poor survival was more pronounced for Native Hawaiian/Pacific Islander women (Model 1, Table 3). Since the fully adjusted model (Model 2) contained fewer cases due to missing data for the additional adjustment variables, we ran the multivariable adjusted models (Model 1) in a complete case analysis, and all results were similar (Supplementary Table S3). We also removed the classifications that were based on genetic ancestry (*n* = 119 Asian participants and *n* = 5 Native Hawaiian/Pacific Islander participants), thereby having more participants assigned as Asian or Native Hawaiian/Pacific Islander not specified and found no appreciable differences (Supplementary Table S4). In analyses of hormone-related factors and family history in relation to survival, we did not observe heterogeneity in the associations between racial and ethnic groups (*p* for heterogeneity≥0.31, Table 4). A notable finding from this analysis was that Hispanic women with a BMI ≥ 30 kg/m^2^ (versus <25) had worse survival (HR = 1.41, 95% CI = 0.99–2.01, *p* for trend=0.06). In a similar comparison among Non-Hispanic White women the HR = 1.15 (95% CI = 1.08–1.23).

**Table 3:** Associations between racial and ethnic groups and overall survival in invasive epithelial ovarian cancer cases.

Epithelial ovarian cancer group	Racial and ethnic group	Cases^c^	Deaths^c^	Model 1^c^	Cases^d^	Deaths^d^	Model 2^d^
		*N*	*N* (%)	HR (95% CI)	*N*	*N* (%)	HR (95% CI)
Total EOC	Non-Hispanic White	12,998	7663 (59.0)	1.00	7663	4767 (62.2)	1.00
	Hispanic	492	285 (57.9)	1.09 (0.96, 1.24)	299	183 (61.2)	1.10 (0.94, 1.29)
	Non-Hispanic Black	267	172 (64.4)	**1.31 (1.12, 1.54)**	154	103 (66.9)	1.23 (0.99, 1.51)
	Asian	697	328 (47.1)	0.89 (0.78, 1.01)	489	248 (50.7)	0.94 (0.80, 1.11)
	Native Hawaiian/Pacific Islander	98	59 (60.2)	**1.58 (1.16, 2.16)**	92	53 (57.6)	**1.47 (1.06, 2.04)**
	Asian or Native Hawaiian/Pacific Islander not specified^b^	127	64 (50.4)	1.14 (0.88, 1.47)	89	47 (52.8)	1.26 (0.93, 1.70)
High grade serous^a^	Non-Hispanic White	8310	5829 (70.1)	1.00	5155	3760 (72.9)	1.00
	Hispanic	332	217 (65.4)	1.01 (0.88, 1.17)	205	144 (70.2)	1.06 (0.89, 1.27)
	Non-Hispanic Black	195	139 (71.3)	**1.29 (1.08, 1.54)**	109	80 (73.4)	1.20 (0.95, 1.52)
	Asian	327	199 (60.9)	0.89 (0.76, 1.06)	242	157 (64.9)	0.92 (0.75, 1.12)
	Native Hawaiian/Pacific Islander	47	41 (87.2)	**2.00 (1.37, 2.92)**	43	37 (86.1)	**1.86 (1.24, 2.80)**
	Asian or Native Hawaiian/Pacific Islander not specified^b^	74	50 (67.6)	1.27 (0.94, 1.71)	50	36 (72.0)	1.32 (0.93, 1.87)
Regional or distant disease	Non-Hispanic White	10,335	6915 (66.9)	1.00	6480	4467 (68.9)	1.00
	Hispanic	394	265 (67.3)	1.10 (0.97, 1.26)	233	171 (73.4)	1.15 (0.97, 1.35)
	Non-Hispanic Black	214	156 (72.9)	**1.36 (1.15, 1.60)**	117	91 (77.8)	**1.30 (1.05, 1.62)**
	Asian	480	271 (56.5)	**0.85 (0.74, 0.98)**	338	206 (61.0)	0.91 (0.77, 1.09)
	Native Hawaiian/Pacific Islander	65	53 (81.5)	**1.73 (1.26, 2.39)**	59	47 (79.7)	**1.64 (1.16, 2.32)**
	Asian or Native Hawaiian/Pacific Islander not specified^b^	104	59 (56.7)	1.14 (0.87, 1.50)	75	44 (58.7)	1.21 (0.88, 1.66)

CI (confidence interval), EOC (epithelial ovarian cancer), HR (hazards ratio).

^a^High-grade serous ovarian carcinoma includes: serous histology grades 2–4; serous histology with missing/unknown grade; endometrioid histology grade 3–4 and poorly differentiated epithelial histology grades 2–4.

^b^Asian or Native Hawaiian/Pacific Islander not specified refers to individuals who were unable to be classified into more specific groups.

^c^Model 1 (multivariable) was adjusted for age (continuous), year of diagnosis (continuous), histotype [high-grade serous (reference), low-grade serous, endometrioid, mucinous, clear cell, other epithelial]; the following variables were modelled as strata terms: study site, age at diagnosis (10-year groups) and stage (localized, regional, distant, unstaged/missing).

^d^Model 2 (fully adjusted) was additionally adjusted for BMI [<25 (reference), 25–29.9, 30+ kg/m^2^], smoking status [never (reference), former, current smoker] and postmenopausal hormone use [never (reference), ever, pre/perimenopausal].

Bold denotes statistical signifi cance *p*<0.05.

**Table 4:** Multivariable HR^a^ and 95% confidence intervals (CI) for the association between hormone-related factors and family history with overall survival among invasive epithelial ovarian cancer patients across racial and ethnic groups.

	Asian *n* = 697	Native Hawaiian/Pacific Islander *n* = 98	Asian or Native Hawaiian/Pacific Islander, not specified^h^ *n* = 127	Non-Hispanic Black *n* = 267	Hispanic *n* = 492	Non-Hispanic White *n* = 12,998	
Exposure	HR (95% CI)	HR (95% CI)	HR (95% CI)	HR (95% CI)	HR (95% CI)	HR (95% CI)	*p* for heterogeneity^i^
Age at menarche (years)							
<12	1.00	1.00	1.00	1.00	1.00	1.00	0.74
12–13	1.20 (0.82, 1.75)	0.81 (0.34, 1.95)	0.78 (0.26, 2.32)	1.13 (0.71, 1.80)	0.85 (0.62, 1.18)	1.02 (0.96, 1.09)	
≥14	1.25 (0.83, 1.86)	0.94 (0.33, 2.66)	1.10 (0.37, 3.34)	1.10 (0.64, 1.88)	**0.66 (0.45, 0.97)**	1.01 (0.94, 1.09)	
*p* for trend^b^	0.29	0.87	0.91	0.67	0.04	0.75	0.35
Oral contraceptive use							
Never	1.00	1.00	1.00	1.00	1.00	1.00	0.99
<5 years	0.93 (0.66, 1.30)	1.00 (0.42, 2.42)	1.24 (0.46, 3.32)	0.86 (0.52, 1.40)	1.01 (0.74, 1.37)	**0.94 (0.88, 0.99)**	
≥5 years	0.96 (0.62, 1.50)	0.65 (0.22, 1.87)	1.07 (0.33, 3.45)	0.88 (0.50, 1.55)	0.87 (0.56, 1.34)	0.96 (0.90, 1.02)	
p for trend^b^	0.74	0.48	0.83	0.60	0.63	0.17	0.99
Parity							
0 live births	1.00	1.00	1.00	1.00	1.00	1.00	0.31
1	0.75 (0.50, 1.14)	0.42 (0.12, 1.49)	0.66 (0.17, 2.55)	0.74 (0.37, 1.47)	0.80 (0.48, 1.34)	1.02 (0.94, 1.12)	
2	0.74 (0.51, 1.08)	0.75 (0.24, 2.32)	0.54 (0.17, 1.74)	1.05 (0.53, 2.08)	1.07 (0.68, 1.68)	0.97 (0.91, 1.04)	
≥3	0.91 (0.63, 1.32)	1.77 (0.63, 4.97)	0.35 (0.07, 1.72)	1.00 (0.54, 1.86)	0.84 (0.56, 1.28)	0.98 (0.92, 1.05)	
*p* for trend^b^	0.61	0.09	0.18	0.62	0.54	0.40	0.37
Tubal ligation							
No	1.00	1.00	1.00	1.00	1.00	1.00	0.97
Yes	1.01 (0.69, 1.48)	0.90 (0.40, 2.01)	1.59 (0.43, 5.89)	1.06 (0.63, 1.79)	0.93 (0.66, 1.31)	0.95 (0.89, 1.02)	
Breastfeeding^c^							
No	1.00	1.00	1.00	1.00	1.00	1.00	0.36
Yes	1.33 (0.91, 1.92)	1.37 (0.63, 2.96)	1.03 (0.20, 5.43)	1.54 (0.89, 2.68)	1.04 (0.73, 1.48)	0.98 (0.92, 1.05)	
Postmenopausal hormone use^d^							
Never	1.00	1.00	1.00	1.00	1.00	1.00	0.55
Ever	0.75 (0.54, 1.05)	1.11 (0.45, 2.74)	0.17 (0.02, 1.42)	0.78 (0.42, 1.45)	0.80 (0.57, 1.14)	**0.89 (0.84, 0.94)**	
Endometriosis							
No	1.00	1.00	1.00	1.00	1.00	1.00	0.48
Yes	1.23 (0.81, 1.88)	0.78 (0.16, 3.71)	0.51 (0.14, 1.83)	0.50 (0.17, 1.46)	1.23 (0.65, 2.32)	0.94 (0.85, 1.04)	
Hysterectomy^e^							
No	1.00	1.00	1.00	1.00	1.00	1.00	0.36
Yes	1.20 (0.81, 1.79)	1.11 (0.42, 2.96)	4.69 (0.40, 55.1)	0.97 (0.59, 1.58)	0.72 (0.50, 1.03)	0.96 (0.90, 1.03)	
BMI, recent^f^							
<25.0 kg/m^2^	1.00	1.00	1.00	1.00	1.00	1.00	0.64
25.0–29.9	0.87 (0.62, 1.22)	0.37 (0.15, 0.91)	1.28 (0.42, 3.90)	1.14 (0.69, 1.90)	1.05 (0.74, 1.48)	0.99 (0.94, 1.06)	
≥30.0	1.12 (0.68, 1.84)	0.58 (0.26, 1.30)	1.84 (0.56, 6.06)	1.27 (0.72, 2.24)	1.41 (0.99, 2.01)	**1.15 (1.08, 1.23)**	
*p* for trend^b^	0.92	0.29	0.32	0.40	0.06	<0.001	0.48
Smoking							
Never	1.00	1.00	1.00	1.00	1.00	1.00	0.53
Former	1.04 (0.71, 1.53)	0.51 (0.24, 1.07)	2.06 (0.61, 7.00)	1.15 (0.64, 2.07)	1.17 (0.81, 1.71)	1.03 (0.98, 1.09)	
Current	0.60 (0.26, 1.34)	0.85 (0.31, 2.31)	1.72 (0.17, 17.3)	1.16 (0.62, 2.16)	0.87 (0.51, 1.49)	**1.13 (1.04, 1.22)**	
Family history of breast cancer							
No	1.00	1.00	1.00	1.00	1.00	1.00	0.99
Yes	0.92 (0.59, 1.45)	1.30 (0.36, 4.66)	1.13 (0.28, 4.52)	0.96 (0.52, 1.78)	0.83 (0.53, 1.29)	**0.93 (0.87, 0.99)**	
Family history of ovarian cancer^g^							
No	1.00	–	–	1.00	1.00	1.00	0.50
Yes	0.87 (0.36, 2.13)	–	–	1.48 (0.61, 3.62)	1.01 (0.59, 1.72)	0.93 (0.83, 1.03)	

BMI (Body Mass Index), CI (confidence interval), HR (Hazard ratio).

^a^Models were adjusted for age (continuous), year of diagnosis (continuous), histotype [high-grade serous (reference), low-grade serous, endometrioid, mucinous, clear cell, other epithelial]; the following variables were modelled as strata terms: study site, age at diagnosis (10-year groups) and stage.

^b^*p* for trend was calculated using the median for that category: age at menarche (years, 10, 12.5, 14); oral contraceptive use (years, 0, 2.5, 5); parity (0 live births, 1, 2, 3); BMI, recent (25 kg/m^2^, 27.5, 30).

^c^Breastfeeding among parous women only.

^d^Postmenopausal hormone use refers to ever use of estrogen only, estrogen plus progesterone and unknown formulation types among postmenopausal women only.

^e^Hysterectomy analysis excludes one study outlier (MAY).

^f^Recent BMI refers to 1 year before the reference date for all sites, except for CON, DOV HAW and OPL (5 years before reference date).

^g^Family history of ovarian cancer estimates could not be calculated for Native Hawaiian/Pacific Islander participants due to small numbers.

^h^Asian or Native Hawaiian/Pacific Islander not specified includes individuals who were unable to be classified into more specific groups.

^i^*p*-value for heterogeneity was calculated using the global Wald test on the interaction terms (race and ethnicity and categorical exposure variable(s), or race and ethnicity and trend exposure variable).

Bold denotes statistical signifi cance *p*<0.05.

## Discussion

Here we observed that overall survival was 1.6 times worse for Native Hawaiian/Pacific Islander and 1.3 times worse for Non-Hispanic Black women with EOC compared with non-Hispanic White women. These survival disparities were apparent for all EOC, within the most common histological subtype, HGSC, and when we restricted to individuals with regional/distant stage at diagnosis. The findings for Native Hawaiian/Pacific Islander women with EOC are comparable to a recent study using SEER data, which also reported the lowest age-standardised 5-year cause-specific survival in serous and distant stage disease for Hawaiian/Pacific Islander women compared with non-Hispanic White and other subgroups of Asian populations [6]. The poorer EOC survival that has been observed for Native Hawaiian/Pacific Islander women extends to other cancer types, including breast [21] and endometrial cancer [22], and is coupled with a higher comorbidity burden compared to White women, which could be an important contributing factor. Healthcare access and physician shortages [23] may contribute to the disparity observed for Native Hawaiian/Pacific Islander women, particularly given that the majority of cases analysed came from a single study on O’ahu, although data on access to treatment were unavailable in the current study. In contrast, an analysis performed in a US military population where individuals had equal access to healthcare observed no significant difference in survival between Pacific Islander and White women [4]. Using other approaches to ascertain healthcare access in future work would be highly valuable.

The opposite direction of survival associations between Native Hawaiian/Pacific Islander and Asian women, when compared to non-Hispanic White women, demonstrates that important survival disparities for Native Hawaiian/Pacific Islander women could be masked if a combined grouping was used, as has been the case until very recently. A similar pattern has been observed in breast cancer [24], emphasising that data disaggregation of Native Hawaiian/Pacific Islander and Asian groups is important to identify priority areas for focused research [7].

Our finding of 1.3-fold poorer survival for non-Hispanic Black women with EOC is equivalent to estimates from the OCWAA consortium [2]. It is estimated that nearly half of the non-Hispanic Black participants in the current study were represented in OCWAA. Disparities in cancer outcomes by racial and ethnic group can be related to structures and systems that disadvantaged subpopulations of the community have historically encountered. The issues are multifactorial, including structural racism, language, and other social determinants of health, including housing and broader neighbourhood context, which can each contribute to disparities among disadvantaged populations [25, 26]. Differential access to treatment [27–29], or adherence to treatment guidelines [29], has been thought to play a major role in disparities between Black and White women with EOC. We also observed that Asian women had a non-significant, 11% better survival compared with non-Hispanic White women, consistent with analyses from the SEER and other US-based populations that included Asian women [3, 4].

Despite known differences in the prevalence of hormone-related factors between racial and ethnic groups [30, 31], there was no heterogeneity observed in the associations between these factors with overall survival across racial and ethnic groups. However, it is possible that the small sample size for some groups (in particular for Native Hawaiian/Pacific Islander participants) was a factor. Notably, the poor survival observed for Hispanic women with a high BMI warrants attention for this subgroup of patients with EOC. Despite a higher prevalence of overweight and obesity in Hispanic populations, studies in patients with breast cancer have not shown significant associations between BMI and survival, with the exception of individuals with BMI ≥ 40 kg/m^2^ [32], although sample sizes have been relatively small [33, 34].

Key strengths of this study included our ability to disaggregate Native Hawaiian and Pacific Islander women from Asian women to study EOC overall survival outcomes while accounting for potentially modifiable factors (postmenopausal hormone use, BMI, and smoking) previously shown to influence EOC survival in studies mostly representing non-Hispanic White women [9, 11, 13], and also observed in Black women with EOC [35]. Limitations were that the racial and ethnic groupings in the current study still represented heterogeneous populations, and future efforts should conduct more granular analyses when possible, in particular to examine specific Asian subpopulations. This heterogeneity would also be reflected in the disparate geographic regions of the participating studies, and there may be different cultural contexts and variations that would be important to consider in future work. Additionally, the current study adjusted for lifestyle factors, however there may be unmeasured confounders or mediators that influence survival, such as comorbidities, treatment received or residual disease after surgery, and these were not available for the current analyses. Prior analysis in the non-Hispanic Black population [2] has shown that some of the variables included in our models as confounders may act as mediators (histotype, stage, smoking, BMI, PMH use). Future work is needed to assess mediators that contribute to observed differences in EOC survival in different racial and ethnic groups. [36] Our dataset lacked information on individual-level or neighbourhood socioeconomic status, insurance status or other social determinants of health, although the highest educational attainment was reported [37] and adjusting for education in the current study did not change the results. We assessed overall survival rather than ovarian cancer-specific survival; therefore, the analysis included some deaths due to other causes. However, EOC is a highly aggressive disease, and of the OCAC studies with cause of death data (representing 38% of deaths), more than 80% were due to ovarian cancer. Lastly, the current analysis evaluated risk factor associations across racial and ethnic groups using the global Wald test to assess heterogeneity in the survival associations; however, this test may be limited for small groups (Native Hawaiian/Pacific Islander women).

Our study revealed poorer overall survival for Native Hawaiian/Pacific Islander women with EOC in a consortium-based study population and provided further evidence demonstrating worse survival outcomes for non-Hispanic Black women. We highlighted that Native Hawaiian/Pacific Islander women with EOC should be considered separately in future analyses to better understand potential survival disparities. New studies, with more contemporary and diverse populations, will be critical to enabling these analyses. Further research and efforts should also focus on understanding the social, economic, or access issues that may be driving this inequity and finding solutions.

## Supplementary information

Supplementary information

## Data Availability

The data used for this analysis will be shared upon approval of a data request by the Ovarian Cancer Association Consortium Data Access Coordinating Committee and participating studies with appropriate human subjects’ approval and data transfer agreements.
