# Supplementary material for: Differences in overall survival for invasive epithelial ovarian cancer by race and ethnicity: results from the Ovarian Cancer Association Consortium
Source: Br J Cancer. 2026 Jun 30;135(7):1057–68. doi: 10.1038/s41416-026-03509-8 (PMC13578740; doi:10.1038/s41416-026-03509-8)
Supplement: Supplementary file 1 [file 41416_2026_3509_MOESM1_ESM.docx]

**Meagher et al. ‘Differences in overall survival for invasive epithelial ovarian cancer by race and ethnicity: results from the Ovarian Cancer Association Consortium.’**

**Supplementary Table S1: Ovarian Cancer Association Consortium (OCAC) primary treatment variable coding and completeness.**

| **OCAC variable name** | **Description** | **Coding** | **Completeness** |
| --- | --- | --- | --- |
| **Prim_treat** | Did the participant have chemotherapy or other systemic treatment as part of their primary treatment? | 1=Yes, 2=No, 8=Unknown | Missing or unknown for 11427/14679 cases (79%)  Missing for 13/18 whole studies |
| **Primary_therapy_outcome** | Primary therapy outcome | CR=Complete response, PR=Partial response, SD=Stable disease, PD=Progressive disease, NK=Not Known | Missing or unknown for 9976/14679 cases (68%)  Missing for 12/18 whole studies |
| **resdx** | Residual Disease | *1 = no macroscopic disease* |  |
|  |  | *2 = macroscopic disease <1 cm* | Missing or unknown for 8903/14679 cases (61%)  Missing for 10/18 whole studies |
|  |  | *3 = macroscopic disease >1 and < 2cm* |  |
|  |  | *4 = macroscopic disease <2 cm (but don't know if < 1 or not)* |  |
|  |  | *5 = macroscopic disease >2 cm (eg debulking laparotomy performed but residual disease was >2 cm)* |  |
|  |  | *6 = macroscopic disease, size unknown* |  |
|  |  | *7 = tumour not resected (eg inoperable; biopsy only)* |  |
|  |  | *8 = macroscopic disease >1 cm (with no further size detail)* |  |
|  |  | *9 = unknown* |  |

**Supplementary Table S2: Year of diagnosis by study**

| Study^a^ | Cases  N | Year of diagnosis (range) | Year of diagnosis, median (IQR) |
| --- | --- | --- | --- |
| TOTAL | 14,679 | 1968-2018 | 2004 (2001-2007) |
| AOV | 277 | 1982-2010 | 2007 (2001-2010) |
| AUS | 1,540 | 2001-2006 | 2004 (2003-2005) |
| BEL | 621 | 1992-2013 | 2010 (2009-2011) |
| CON | 385 | 1998-2003 | 2001 (2000-2002) |
| DOV | 1,125 | 2002-2009 | 2005 (2003-2007) |
| HAW | 424 | 1992-2008 | 2001 (1996-2004) |
| HOP | 709 | 2003-2008 | 2005 (2004-2007) |
| LAX | 352 | 1984-2012 | 2002 (1999-2005) |
| MAY | 1,463 | 1999-2018 | 2009 (2005-2013) |
| MSK | 505 | 1998-2009 | 2004 (2002-2007) |
| NEC | 1,255 | 1992-2008 | 2001 (1996-2004) |
| NJO | 235 | 2003-2008 | 2005 (2004-2006) |
| OPL | 798 | 2011-2015 | 2013 (2012-2014) |
| SEA | 1,469 | 1968-2013 | 2003 (1998-2008) |
| STA | 448 | 1996-2001 | 1998 (1997-1999) |
| UCI | 429 | 1989-2003 | 2000 (1995-2002) |
| UKO | 743 | 1988-2009 | 2006 (2004-2007) |
| USC | 1,901 | 1991-2009 | 2000 (1995-2004) |
| IQR (interquartile range) | | | |

^a^Study acronyms: AOV (Alberta Ovarian Tumor Types study, Canada, PubMed ID: 25349970); AUS (Australian Ovarian Cancer and Australian Cancer Study, PubMed ID:17721999); BEL (University Hospitals Leuven, Department of Gynaecological Oncology, Tumor biobank, Belgium); CON (Connecticut Ovary Study, PubMed ID:16985038); DOV (Diseases of the Ovary and their Evaluation Study, PubMed ID: 23065074); HAW (Hawaii Ovarian Cancer Case-Control Study, PubMed ID:18667686); HOP (Hormones and Ovarian Cancer PrEdiction, PubMed ID: 22252409); LAX (Women's Cancer Program at the Samuel Oschin Comprehensive Cancer Institute, PubMed ID: 22253144); MAY (Mayo Clinic Ovarian Cancer Case Control Study, PubMed ID:18381459); MSK (Memorial Sloan Kettering Cancer Center Gynecologic Tissue Bank); NEC (New England Case-Control Study of Ovarian Cancer, PubMed ID:15994977); NJO (New Jersey Ovarian Cancer Study, PubMed ID:21943063); OPL (Ovarian Cancer Prognosis and Lifestyle study, Australia, PubMed ID: 33749900); SEA (UK Studies of Epidemiology and Risk Factors in Cancer Heredity (SEARCH) Ovarian Cancer Study, PubMed ID: 16774946); STA (Genetic Epidemiology of Ovarian Cancer, PubMed ID:15383404); UCI (University of California, Irvine Ovarian Cancer Study, PubMed ID:10667470); UKO (United Kingdom Ovarian Cancer Population Study, PubMed ID: 21074968); USC (Los Angeles County Case-Control Studies of Ovarian Cancer, PubMed ID:11821246).

**Supplementary Table S3: Associations between racial and ethnic group and overall survival in invasive epithelial ovarian cancer cases, original population and complete case analysis.**

| **Epithelial ovarian cancer group** | **Racial and ethnic group** | **Cases^a^** | **Deaths^a^** | **Model 1^a^** | **Cases^b^** | **Deaths^b^** | **Model 1^b^ (complete case analysis)** |
| --- | --- | --- | --- | --- | --- | --- | --- |
|  |  | **N** | **N (%)** | **HR (95% CI)** | **N** | **N (%)** | **HR (95% CI)** |
| Total EOC | Non-Hispanic White | 12,998 | 7663 (59.0) | 1.00 | 7663 | 4767 (62.2) | 1.00 |
|  | Hispanic | 492 | 285 (57.9) | 1.09 (0.96, 1.24) | 299 | 183 (61.2) | 1.11 (0.95, 1.30) |
|  | Non-Hispanic Black | 267 | 172 (64.4) | **1.31 (1.12, 1.54)** | 154 | 103 (66.9) | **1.28 (1.04, 1.57)** |
|  | Asian | 697 | 328 (47.1) | 0.89 (0.78, 1.01) | 489 | 248 (50.7) | 0.91 (0.77, 1.07) |
|  | Native Hawaiian/Pacific Islander | 98 | 59 (60.2) | **1.58 (1.16, 2.16)** | 92 | 53 (57.6) | **1.52 (1.09, 2.11)** |
|  | Asian or Native Hawaiian/Pacific Islander not specified^c^ | 127 | 64 (50.4) | 1.14 (0.88, 1.47) | 89 | 47 (52.8) | 1.20 (0.89, 1.63) |
| High grade serous^d^ | Non-Hispanic White | 8,310 | 5829 (70.1) | 1.00 | 5155 | 3760 (72.9) | 1.00 |
|  | Hispanic | 332 | 217 (65.4) | 1.01 (0.88, 1.17) | 205 | 144 (70.2) | 1.10 (0.92, 1.31) |
|  | Non-Hispanic Black | 195 | 139 (71.3) | **1.29 (1.08, 1.54)** | 109 | 80 (73.4) | 1.26 (0.99, 1.60) |
|  | Asian | 327 | 199 (60.9) | 0.89 (0.76, 1.06) | 242 | 157 (64.9) | 0.89 (0.73, 1.09) |
|  | Native Hawaiian/Pacific Islander | 47 | 41 (87.2) | **2.00 (1.37, 2.92)** | 43 | 37 (86.1) | **1.98 (1.32, 2.97)** |
|  | Asian or Native Hawaiian/Pacific Islander not specified^c^ | 74 | 50 (67.6) | 1.27 (0.94, 1.71) | 50 | 36 (72.0) | 1.29 (0.91, 1.82) |
| Regional or distant disease | Non-Hispanic White | 10,335 | 6915 (66.9) | 1.00 | 6480 | 4467 (68.9) | 1.00 |
|  | Hispanic | 394 | 265 (67.3) | 1.10 (0.97, 1.26) | 233 | 171 (73.4) | 1.16 (0.99, 1.37) |
|  | Non-Hispanic Black | 214 | 156 (72.9) | **1.36 (1.15, 1.60)** | 117 | 91 (77.8) | **1.35 (1.09, 1.68)** |
|  | Asian | 480 | 271 (56.5) | **0.85 (0.74, 0.98)** | 338 | 206 (61.0) | 0.88 (0.74, 1.05) |
|  | Native Hawaiian/Pacific Islander | 65 | 53 (81.5) | **1.73 (1.26, 2.39)** | 59 | 47 (79.7) | **1.71 (1.21, 2.41)** |
|  | Asian or Native Hawaiian/Pacific Islander not specified^c^ | 104 | 59 (56.7) | 1.14 (0.87, 1.50) | 75 | 44 (58.7) | 1.16 (0.85, 1.59) |
| Abbreviations: CI (confidence interval); EOC (epithelial ovarian cancer); HR (hazards ratio). | | | | | | | |
| ^a^ Multivariable adjusted model (from Table 3) was adjusted for age (continuous) year of diagnosis (continuous), histotype [high-grade serous (reference), low-grade serous, endometrioid, mucinous, clear cell, other epithelial]; the following variables were modelled as strata terms: study site, age at diagnosis (10-year groups) and stage. | | | | | | | |
| ^b^ Multivariable adjusted models were run in a complete case analysis. Multivariable models were adjusted for age and year of diagnosis (both continuous), histotype [high-grade serous (reference), low-grade serous, endometrioid, mucinous, clear cell, other epithelial]; study site, age at diagnosis (10-year groups) and stage were modelled as strata terms. The complete case population was defined as participants with complete data on postmenopausal hormone use, body mass index and smoking. | | | | | | | |
| ^c^ Asian or Native Hawaiian/Pacific Islander not specified includes individuals who were unable to be classified into more specific groups. | | | | | | | |
| ^d^ High-grade serous ovarian carcinoma includes: serous histology grades 2-4; serous histology with missing/unknown grade; endometrioid histology grade 3-4 and poorly differentiated epithelial histology grades 2-4. | | | | | | | |

**Supplementary Table S4: Associations between racial and ethnic groups and overall survival in invasive epithelial ovarian cancer cases, including only self-reported race (no genetic ancestry data).**

| Epithelial ovarian cancer group | Racial and ethnic group | Cases^a^ | Deaths^a^ | Model 1^a^ | Cases^b^ | Deaths^b^ | Model 2^b^ |
| --- | --- | --- | --- | --- | --- | --- | --- |
|  |  | N | N (%) | HR (95% CI) | N | N (%) | HR (95% CI) |
| Total EOC | Non-Hispanic White | 12,998 | 7663 (59.0) | 1.00 | 7663 | 4767 (62.2) | 1.00 |
|  | Hispanic | 492 | 285 (57.9) | 1.09 (0.96, 1.24) | 299 | 183 (61.2) | 1.10 (0.93, 1.28) |
|  | Non-Hispanic Black | 267 | 172 (64.4) | **1.31 (1.12, 1.54)** | 154 | 103 (66.9) | 1.23 (0.99, 1.51) |
|  | Asian | 578 | 281 (48.6) | 0.89 (0.76, 1.03) | 416 | 218 (52.4) | 0.95 (0.80, 1.14) |
|  | Native Hawaiian/Pacific Islander | 93 | 55 (59.1) | **1.53 (1.11, 2.10)** | 90 | 52 (57.8) | **1.47 (1.05, 2.06)** |
|  | Asian or Native Hawaiian/Pacific Islander not specified^c^ | 251 | 115 (45.8) | 1.04 (0.86, 1.27) | 164 | 78 (47.6) | 1.08 (0.85, 1.38) |
| High grade serous^d^ | Non-Hispanic White | 8310 | 5829 (70.1) | 1.00 | 5155 | 3760 (72.9) | 1.00 |
|  | Hispanic | 332 | 217 (65.4) | 1.01 (0.88, 1.17) | 205 | 144 (70.2) | 1.06 (0.89, 1.27) |
|  | Non-Hispanic Black | 195 | 139 (71.3) | **1.29 (1.08, 1.54)** | 109 | 80 (73.4) | 1.20 (0.95, 1.52) |
|  | Asian | 265 | 170 (64.2) | 0.89 (0.74, 1.07) | 206 | 137 (66.5) | 0.94 (0.75, 1.16) |
|  | Native Hawaiian/Pacific Islander | 45 | 39 (86.7) | **2.00 (1.34, 2.97)** | 42 | 36 (85.7) | **1.88 (1.23, 2.85)** |
|  | Asian or Native Hawaiian/Pacific Islander not specified^c^ | 138 | 81 (58.7) | 1.13 (0.90, 1.42) | 87 | 57 (65.5) | 1.11 (0.84, 1.47) |
| Regional or distant disease | Non-Hispanic White | 10,335 | 6915 (66.9) | 1.00 | 6480 | 4467 (68.9) | 1.00 |
|  | Hispanic | 394 | 265 (67.3) | 1.10 (0.97, 1.26) | 233 | 171 (73.4) | 1.15 (0.98, 1.35) |
|  | Non-Hispanic Black | 214 | 156 (72.9) | **1.36 (1.15, 1.60)** | 117 | 91 (77.8) | **1.30 (1.05, 1.62)** |
|  | Asian | 382 | 229 (60.0) | 0.85 (0.72, 1.00) | 278 | 179 (64.4) | 0.93 (0.77, 1.14) |
|  | Native Hawaiian/Pacific Islander | 61 | 49 (80.3) | **1.68 (1.20, 2.35)** | 58 | 46 (79.3) | **1.65 (1.16, 2.35)** |
|  | Asian or Native Hawaiian/Pacific Islander not specified^c^ | 206 | 105 (51.0) | 1.02 (0.83, 1.25) | 136 | 72 (52.9) | 1.03 (0.81, 1.32) |
| Abbreviations: CI (confidence interval); EOC (epithelial ovarian cancer); HR (hazards ratio). | | | | | | | |
| ^a^ Model 1 (multivariable) was adjusted for age (continuous), year of diagnosis (continuous), histotype [high-grade serous (reference), low-grade serous, endometrioid, mucinous, clear cell, other epithelial]; the following factors were modelled as strata terms: study site, age at diagnosis (10-year groups) and stage (localized, regional, distant, unstaged/missing). | | | | | | | |
| ^b^ Model 2 (fully adjusted) was additionally adjusted for BMI [<25(reference), 25-29.9, 30+ kg/m^2^], smoking status [never(reference), former, current smoker] and postmenopausal hormone use [never (reference), ever, pre/perimenopausal]. | | | | | | | |
| ^c^ Asian or Native Hawaiian/Pacific Islander not specified includes individuals who were unable to be classified into more specific groups. | | | | | | | |
